# Physics-informed reinforcement learning for motion control of a fish-like swimming robot

**DOI:** 10.1038/s41598-023-36399-4

**Published:** 2023-07-03

**Authors:** Colin Rodwell, Phanindra Tallapragada

**Affiliations:** Department of Mechanical Engineering, Clemson University, Clemson, SC 29634 USA

**Keywords:** Mechanical engineering, Fluid dynamics, Nonlinear phenomena

## Abstract

Motion control of fish-like swimming robots presents many challenges due to the unstructured environment and unmodelled governing physics of the fluid–robot interaction. Commonly used low-fidelity control models using simplified formulas for drag and lift forces do not capture key physics that can play an important role in the dynamics of small-sized robots with limited actuation. Deep Reinforcement Learning (DRL) holds considerable promise for motion control of robots with complex dynamics. Reinforcement learning methods require large amounts of training data exploring a large subset of the relevant state space, which can be expensive, time consuming, or unsafe to obtain. Data from simulations can be used in the initial stages of DRL, but in the case of swimming robots, the complexity of fluid–body interactions makes large numbers of simulations infeasible from the perspective of time and computational resources. Surrogate models that capture the primary physics of the system can be a useful starting point for training a DRL agent which is subsequently transferred to train with a higher fidelity simulation. We demonstrate the utility of such physics-informed reinforcement learning to train a policy that can enable velocity and path tracking for a planar swimming (fish-like) rigid Joukowski hydrofoil. This is done through a curriculum where the DRL agent is first trained to track limit cycles in a velocity space for a representative nonholonomic system, and then transferred to train on a small simulation data set of the swimmer. The results show the utility of physics-informed reinforcement learning for the control of fish-like swimming robots.

## Introduction

The locomotion of fish and other aquatic swimmers has many desirable characteristics such as energy efficiency, agility, and stealth^1,2^, which have inspired the design of many biomimetic robots. Designs for fish-like robots include those that are assemblages, rigid links actuated by motors imitating the motion of tails and fins^3^, motor-driven flexible links^4,5^, elongated snake and eel like robots^6,7^, soft robots making use of dielectric elastomers, electroactive polymers or fluidic elastomer actuators^8–10^, or robots with internal reaction wheels^11,12^. In all such designs, the small size of the fish-like robots and the resulting constraints on actuation and power require that the robots harness the fluid structure and fluid–structure interaction for efficient and agile motion. The control of such robots therefore requires modeling of the fluid–structure interaction that determines the forces and moments on a robot. Motion control of robots, either physical or simulated with high fidelity, moving in unstructured environments with complex or unmodelled governing physics has traditionally presented many challenges. Low-fidelity models using simplified formulas for drag and lift forces lead to models that are amenable for control, but do not capture key physics that can play an especially important role in the swimming of small-scale robots with limited actuation. Deep Reinforcement Learning (DRL) holds considerable promise for motion control of robots with complex dynamics, such as swimming robots. Reinforcement learning can be particularly useful when precise governing models are absent^13–15^.

Reinforcement learning requires the acquisition of large amounts of data from the robotic system in the form of states, control actions, and the resulting state updates. Such data can be acquired either through experiments, field tests, or simulations of the robot and its interaction with the environment. In the case of mobile robots, experiments and field tests could prove to be very expensive or unsafe to the robot. As a result, simulations play an important role in reinforcement learning for robotics^16–18^. Simulations can in theory generate large amounts of data at low cost, exploring a large subset of the state space that is challenging to explore in experiments. This is true in the case of robotic manipulators, inverted pendulums and other toy systems whose physics is well understood and can be efficiently and quickly computed. However, where the governing physics is complex or the state space is high-dimensional with possibly discontinuous dynamics, high fidelity simulators are often impractical and significant model reduction or the use of surrogate models is essential^18^.

The control of swimming robots is an important example where the governing physics is complex enough that reinforcement learning should be a tool of choice for control, but the dynamics of the fluid-structure interaction can be challenging to simulate. While reinforcement learning is being widely applied in many areas of robotics^14,19^ and fluid control^20,21^, swimming robots have for the most part not received much attention. A few notable recent exceptions employed reinforcement learning for tasks such as predicting efficient schooling configurations for pairs of swimmers^22^ or larger schools^23^, gait generation^24^, or efficient start and escape patterns^25^ . However, important problems related to mobile robotics such as station keeping, velocity tracking under disturbances, or path tracking have not been addressed in the area of fish-like swimming robots due to the computational challenges of running a large number of high-fidelity simulations.

We address this challenge with curriculum learning^26,27^ and transfer learning^28^, in the context of a swimming robot. It has been observed that when applying reinforcement learning using the Deterministic Policy Gradient (DPG) algorithm for complex tasks, much of computational time is spent going from the initialized random policy to an intermediate policy that, while sub-optimal, has qualitative similarities to the optimal policy^26–28^. Going from this policy to the optimal policy is often very fast. The intermediate policy does not have to be trained using high-fidelity state data, and can instead be cheaply trained using lower-fidelity simulations or models, before being transferred to a higher-fidelity environment to complete the final steps of training. Efficient multi-model training has been demonstrated in fluids for flow control^29^, and here we extend it to the swimming problem. Here we utilize knowledge of the physics of fish-like swimming, specifically two important qualitative features of swimming, to estimate these intermediate policies, from which finding an optimal policy is faster. The first feature is that fish-like propulsion is enabled by periodic ‘tail beating’ (for carangiform fish) or body undulations (anguiliform fish)^30,31^, at least in steady state motion. In a suitable reduced velocity space this feature can be modelled by limit cycles created by periodic forcing. A second surprising feature is that swimming by a hydrofoil, which resembles a cross-section of a fish-like body, can be approximately modelled as motion of a nonholonomically constrained system. This surprising feature arises because the Kutta condition, which creates vorticity at sharp corners of a body moving in an otherwise approximately inviscid fluid, acts as a nonholonomic constraint on a swimming hydrofoil^32,33^.

The swimming robot in this paper is modeled as a free-swimming Joukowski hydrofoil, propelled and steered by an internal reaction wheel^11,12^. A particularly simple surrogate model that emulates the dynamics of such a swimmer is a nonholonomic system known as the Chaplygin sleigh. Periodic torque on the Chaplygin sleigh produces ‘figure-8’ limit cycles in the velocity space, which are similar in structure to the observed limit cycles of swimmers. Analytical approximations for these limit cycles as a function of the forcing frequency and amplitude of the torque, as well as the inverse dynamics problem of finding the periodic torque to generate a limit cycle, have been shown in^34^. Torques for steering the Chaplygin sleigh^12,34^ and path tracking for the Chaplygin sleigh using a vector pursuit method were demonstrated in^35^. Since we are interested in transfer learning using the low-dimensional model of the Chaplygin sleigh, we revisit the problem of simultaneous velocity tracking and steering using a reinforcement learning framework. A DPG agent^36,37^ is trained to generate the action (torque) to steer a Chaplygin sleigh which has parameters that have been fit to match the dynamics of the swimming Joukowski foil. The agent is trained on the Chaplygin sleigh model to track a limit cycle in the velocity space at a specified translational velocity. This DPG agent of the Chaplygin sleigh is then transferred to a fluid simulation for training to steer the hydrofoil and track a speed. This second training requires fewer simulations to fine tune the DPG agent’s precision in tracking a reference velocity. This step-by-step curriculum reinforcement learning circumvents the problem of high computational time to simulate the physics of the system^26,27^ and allows a way to *imprint qualitative physics* into a DPG agent.

This paper sets forth a framework for using physics-informed surrogate models to train a DPG agent which is then subsequently trained using data generated from fast simulations of fluid–robot interaction. A very high fidelity computational method need not be used in the second step for two reasons: going from an intermediate sub-optimal policy to an optimal policy can be slowed down significantly, but more importantly the computation of the fluid–robot interaction is intended to be another intermediate step before actual experiments. The paper is organized as follows: in Section “Nonholonomic constraints: Chaplygin sleigh and the Joukowski foil in an inviscid fluid” a short review of nonholonomic constraints with particular reference to the Chaplygin sleigh is provided and the Kutta condition on a Joukowski foil is shown to be formally similar to this constraint. In Section “Periodic forcing and limit cycles in reduced velocity space” limit cycles are shown to exist via simulations in a reduced velocity space for the Chaplygin sleigh and a hydrofoil excited by a periodic torque. Here we make use of a panel method to simulate the motion of the hydrofoil. In Section “Parameter estimation for the Surrogate model” we select two sets of Chaplygin sleigh parameters that model the swimming hydrofoil at different translational velocities. In Section “Reinforcement learning” we describe the reinforcement learning framework with a curriculum to enable path tracking by a Chaplygin sleigh and the transfer of this skill to the simulated swimming hydrofoil.

## Nonholonomic constraints: Chaplygin sleigh and the Joukowski foil in an inviscid fluid

### Chaplygin sleigh

**Figure 1:**
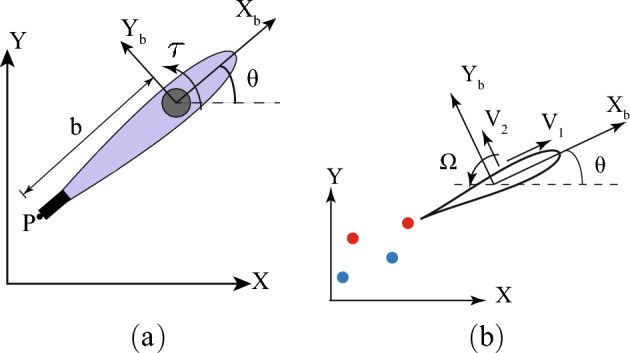
(**a**) A Chaplygin sleigh shaped as a Joukowski foil with a no slip constraint at P in the transverse ($Y_b$) direction. The internal reaction wheel is shown by the grey circle. (**b**) A Joukowski foil with singular distributions of vorticity (red circles corresponding to positive (counterclockwise) vorticity, and blue circles corresponding to negative (clockwise) vorticity) in an otherwise inviscid flow.

The Chaplygin sleigh^38^ or cart, shown in Fig. 1, has a knife edge or a small inertia-less wheel at the rear at point *P* and is supported on a single castor or wheel at the front that allows motion in any direction. The sleigh is also assumed to have an internal reaction wheel whose angular acceleration can apply a torque $\tau$ to the sleigh. The configuration manifold of the physical system is $Q = SE(2)\times S^1$. Because the rotor angle and angular velocity are not coupled with the rest of the system, we eliminate the rotor coordinate except for the torque $\tau$, reducing the system to $Q = SE(2)$. This is parameterized locally by $q = (x,y,\theta )$, where (*x*, *y*) denote the position of the sleigh center of mass and $\theta$ denotes its fixed frame angle relative to the horizontal. The generalized velocities are $\dot{q} = (\dot{x}, \dot{y}, \dot{\theta })$. The tangent space to *Q* at *q* is denoted by $T_q Q \cong \mathbb {R}^3$ and spanned by combinations of the generalized velocities; the standard basis being $\{[1, 0, 0], [0,1,0], [0,0,1]\}$. These three basis vectors are respectively translations $\dot{x}$, $\dot{y}$ and rotation $\dot{\theta }$. The spatial frame is denoted by $\mathscr {F}_S$ with axes $X-Y$ and the body frame, which is collocated at the mass center *C* and rotated by the yaw angle $\theta$ with respect to the spatial frame, is denoted by $\mathscr {F}_B$ with axes $X_b-Y_b$. The velocity $(\dot{x}, \dot{y})$ in the spatial frame transforms to velocity (*u*, *v*) in the body frame as $[u~ v]^{\intercal } = \textbf{R}(\theta )\cdot [\dot{x} ~\dot{y}]^{\intercal }$, where $\textbf{R}(\theta )$ is the rotation matrix.

We assume that the rear wheel at *P* prevents slipping in the transverse $(Y_b)$ direction but rolls freely in the longitudinal direction along $(X_b)$. While $dim(T_qQ) = 3$, the velocity constraint at point *P* is given by

1 $$\begin{aligned} -\sin {\theta } \dot{x} + \cos {\theta } \dot{y} - b \dot{\theta } = 0 \end{aligned}$$

or in the body frame $$v - b \dot{\theta } = 0$$. In terms of the standard basis for $$T_q Q$$, the velocity of sleigh is restricted to lie in the subspace $$W = span\{[\cos {\theta },\sin {\theta }, 0]^\intercal ,[-b\sin {\theta }, b\cos {\theta },1]^\intercal \}$$, with the complementary subspace being defined by (1), $$W^{\perp } = span\{-\sin {\theta }, \cos {\theta }, -b \}$$. Physically, this means that the allowable motions are such that the sleigh can translate along its longitudinal $$(X_b)$$ direction and have no spin velocity i.e. in the fixed frame the velocity of the center of the sleigh is $$\cos {\theta }\dot{x} + \sin {\theta }\dot{y}$$, or if the spin angular velocity of the sleigh is $$\dot{\theta }$$ and the constraint point does not translate, then the center of the sleigh can only translate with $$(\dot{x} = -b\sin {\theta }\dot{\theta }, \dot{y} = b\cos {\theta }\dot{\theta })$$. The distribution (a smooth assignment of a subspace of the tangent space at each $$q \in Q$$) $$D(q) = \{w\in W(q) \subset T_q Q\}$$ is nonholonomic if and only if *D*(*q*) is closed under the Jacobi-Lie brackets of the vector fields in *D*(*q*). The Lie-bracket of two vector fields $$w_1$$ and $$w_2$$ is formally defined as $$[w_1,w_2] = (\nabla w_2) \cdot w_1 - (\nabla w_1) w_2$$. Setting the vectors $$w_1 $$ and $$w_2$$ as the basis vectors defining the span of *W* respectively, their Lie bracket yields $$[w_1, w_2] = [-\sin {\theta }, \cos {\theta }, 0]^\intercal \notin W$$ showing that the constraint (1) is nonholonomic, see^33^ for further details.

### Nonholonomic constraints and swimming in an inviscid fluid

Nonholonomic constraints on the locomotion of a body in a fluid are easiest to realize if one considers the motion of a body with corners in an inviscid fluid. One such common example of a body relevant to both flight and fish-like swimming is the motion of a Joukowski foil whose geometry is described by mapping its boundary from a circle of radius $r_c$ centered at the origin in the mapped plane through the Joukowski transformation

2 $$\begin{aligned} z = F(\zeta ) = \zeta + \zeta _c + \frac{a^2}{\zeta +\zeta _c}, \end{aligned}$$

where $\zeta _c \in \mathbb {C}$ and $a \in \mathbb {R}$ are geometric parameters. We refer to the plane of the foil’s motion as the foil plane and the plane of the circle’s motion as the circle plane. For the symmetrical shape of the foil shown in Fig. 1b, $\zeta _c \in \mathbb {R}$. The pre-image of the sharp trailing edge of the foil is given by $\zeta _t = a-\zeta _c$. We assume the fluid could contain singular distributions of vorticity in the form of *N* point vortices as shown in Fig. 1b. The motion of the fluid is governed by a linear superposition of potential functions. Following Milne-Thomson^39^, the complex potential $W(z) = w(\zeta )$ describing the velocity of the fluid in a body frame of reference ($X_b-Y_b$) (see Fig. 1b) may be decomposed in terms of its dependence on the translation of the foil, the rotation of the foil, and each of the *N* point vortices in the form

$$\begin{aligned} w(\zeta ) = W(z) = V_1 w_1(\zeta ) + V_2 w_2(\zeta ) + \Omega w_3(\zeta ) + \sum \limits _{n=1}^N w^n_v(\zeta ). \end{aligned}$$

Here $w_1$, $w_2$ and $w_3$ are the rigid-body (Kirchoff) potential functions due to the translation of the foil in the $X_b$ and $Y_b$ directions and rotation about the out-of-plane *Z* axis, respectively. The potential function $w^n_v(\zeta )$ due to the *n*th point vortex with circulation $\Gamma _n$ located at $\zeta _n$ outside a circular cylinder can be constructed according to the *Milne–Thomson circle theorem*^39^ in terms of an image vortex of circulation $-\Gamma _n$ located inside the cylinder at $r_c^2 / \bar{\zeta _n}$. Thus $w^n_v(\zeta ) = \frac{\Gamma _n}{2\pi \imath } \left( \log {(\zeta - \zeta _n)} - \log {\left( \zeta -\frac{r_c^2}{\bar{\zeta _n}}\right) }\right)$ .

The image vortex inside the cylinder introduces a net circulation around the cylinder, consistent with Kelvin’s circulation theorem. This development of net circulation around the foil introduces a lift force on the foil and is essential to its propulsion. The complex velocity of the fluid in the foil plane in a body fixed frame is related to the complex velocity of the fluid in the circle plane by the equation,

3 $$\begin{aligned} \frac{dW}{dz} = \frac{dw}{dz}\frac{dz}{d\zeta } = \frac{dw}{dz}\frac{1}{F'(\zeta )} \end{aligned}$$

where $F'(\zeta ) = 1 - \frac{a^2}{(\zeta +\zeta _c)^2}$. Since the fluid has been modeled to be inviscid, the boundary conditions on the body of the foil allow the fluid to slip along the surface. An additional constraint on the velocity of the fluid is necessitated by the geometry of the foil. The pre-image of the trailing edge of the foil is a singularity of the Joukowski transformation, i.e., the derivative $F'(\zeta _t) =0$. It can be seen from Eq. (3) that the complex velocity of the fluid in the circle plane has to be zero to ensure that the velocity of the fluid at the trailing edge of the foil in the foil plane does not become undefined; this is the Kutta condition. The Kutta condition requires that at the pre-image $\zeta _t$ of the trailing edge,

$$\begin{aligned} \frac{dw}{d\zeta }\bigg |_{\zeta = \zeta _t} = V_1\frac{dw_1}{d\zeta }\bigg |_{\zeta = \zeta _t} + V_2\frac{dw_2}{d\zeta }\bigg |_{\zeta = \zeta _t} +\Omega \frac{dw_3}{d\zeta }\bigg |_{\zeta = \zeta _t} + \sum _{1}^{n=N}\frac{dw_v^n}{d\zeta }\bigg |_{\zeta = \zeta _t} = 0. \end{aligned}$$

A formal calculation shows that $\frac{dw_1}{d\zeta }\bigg |_{\zeta = \zeta _t} = 0$ and $\frac{dw_2}{d\zeta }\bigg |_{\zeta = \zeta _t} = -2$. The term $\frac{1}{2}\frac{dw_3}{d\zeta }\bigg |_{\zeta = \zeta _t}$, subsequently denoted by $-b$, is a constant that is determined by the numerical values of the parameters *a* and $\zeta _c$. Denoting the velocity of the fluid at the trailing edge due to the distribution of point vortices $u_v = \frac{1}{2}\sum _{1}^{n=N}\frac{dw_v^n}{d\zeta }\bigg |_{\zeta = \zeta _t}$, the Kutta condition can be re-written as

4 $$\begin{aligned} -\dot{x}\sin {\theta } + \dot{y}\cos {\theta } - b \dot{\theta } = u_v. \end{aligned}$$

Equation (4) constrains the velocity of the foil and it is an *affine-nonholonomic* constraint. This condition constrains the velocity in the system’s phase space to an affine distribution *A* of the form $A(q) = \{w-w_0 \in W_e(q) | w_0 = [-u_v\sin {\theta }, u_v\cos {\theta }, 0]^\intercal \}$, where $W_e(q) = span \{W, V^1, \ldots ,V^{2N} \}$ is the subspace containing the allowable vector fields $w_1$ and $w_2$ associated with the motion of the foil and the vector field $V^1, \ldots , V^{2N}$ being the velocities of each of the *N* point vortices. This is an affine-distribution not simply on *SE*(2) but on the Cartesian product of *SE*(2) with configuration manifold $\mathbb {R}^{2N}$ for the vortices. The vector fields $V^1, \ldots ,V^{2N}$ represent translational velocities of the vortices, potentially dependent on $(x,y,\theta )$, that are compatible with the constraint when the position and orientation of the foil are fixed.

## Periodic forcing and limit cycles in reduced velocity space

### Limit cycles in reduced velocity space of the Chaplygin sleigh

The equations of motion of the Chaplygin sleigh can be calculated in a straightforward manner. Here we will assume that the Chaplygin sleigh experiences viscous resistance as it moves on the ground and that it is actuated by periodic torque generated by the periodic oscillation of the reaction wheel. The Lagrangian of the system is $\mathscr {L}= \frac{1}{2}m(\dot{x}^2 + \dot{y}^2) + \frac{1}{2}I_c\dot{\theta }^2$ where *m* is the mass of the sleigh, *I* is its moment of inertia, and *x* and *y* are the coordinates of the center of mass. Assuming a viscous resistive force to motion described by the Rayleigh dissipation function $\mathscr {R}=\frac{1}{2}(c_{u}(\dot{x}\cos \theta +\dot{y}\sin \theta )^{2} + c_{\omega }\dot{\theta }^{2})$, where $c_u$ and $c_{\omega }$ are viscous damping coefficients for the translational and rotational velocity respectively. The Euler–Lagrange equations are

5 $$\begin{aligned} \frac{d}{dt}\left( \frac{\partial \mathscr {L}}{\partial \dot{q}^i}\right) -\frac{\partial \mathscr {L}}{\partial q^i}= B_{ij}\lambda _j-\frac{\partial \mathscr {R}}{\partial \dot{q}^i}+\tau _i(t), \end{aligned}$$

where $B = [-\sin {\theta }, \cos {\theta }, -b]$ (obtained from $W^{\perp }$, the complementary space of *W*), $\lambda _j$ is the Lagrange multiplier for each *j* constraint and $\tau _{i}$ are any external forces or torques acting on sleigh. Here *i* varies from 1 to 3 and $j=1$, so only $\tau _1$ is nonzero and consequently it is denoted henceforth as just $\tau$. The equations can be transformed to a body frame using $[u~ v]^{\intercal } = \textbf{R}\cdot [\dot{x} ~\dot{y}]^{\intercal }$ to obtain the dimensionless *reduced velocity* equations

6 $$\begin{aligned} \dot{u}&= b \omega ^2-\frac{c_u}{m} u \end{aligned}$$

7 $$\begin{aligned} \dot{\omega }&= \frac{\tau -m b \omega u-c_{\omega } \omega }{I+m b^2}, \end{aligned}$$

where $u=\dot{x}\cos \theta +\dot{y}\sin \theta$ is the translational velocity at the constraint. Due to the nonholonomic constraint, the evolution of the velocities is governed by two equations instead of three. The evolution of the configuration variables is given by

8 $$\begin{aligned} \dot{\theta }&= \omega \end{aligned}$$

9 $$\begin{aligned} \dot{x}&= u\cos {\theta } - \omega b\sin {\theta } \end{aligned}$$

10 $$\begin{aligned} \dot{y}&= u\sin {\theta } + \omega b\cos {\theta }. \end{aligned}$$

**Figure 2:**
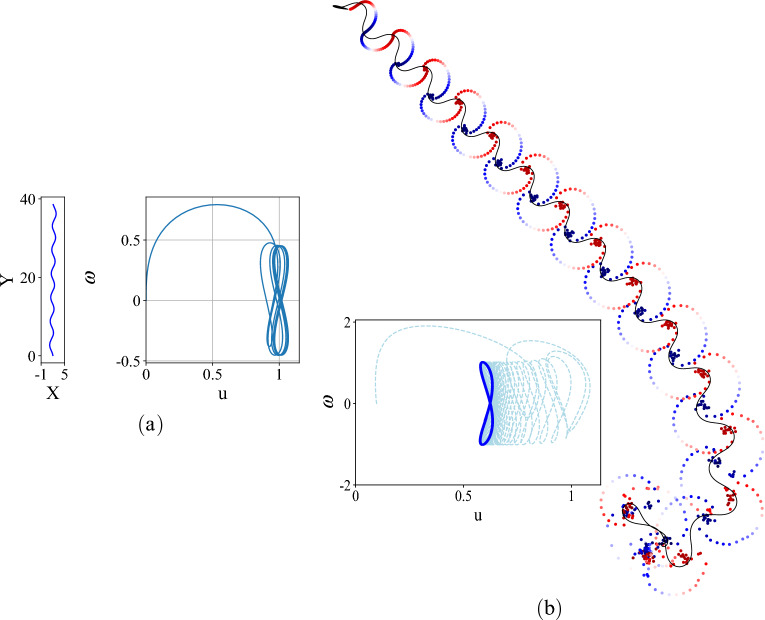
(**a**) (left) A sample serpentine trajectory for the Chaplygin sleigh where the mean converges to a straight line. (right) In the reduced velocity space the trajectory converges to a ‘figure-8’ limit cycle in $(u,\omega )$. (**b**) A sample trajectory of the simulated swimmer starting from rest when forced by a periodic torque $\tau =A \sin {\omega t}$. The inset figure shows convergence to a limit cycle in the reduced velocity, $(u,\omega )$, space that is similar to the that of the Chaplygin sleigh, indicating similar underlying dynamics. The velocity is scaled into body lengths per second ([BL/s]). The swimmer moves along a serpentine path (in black) with the average path converging to a straight line.

When the torque due to the reaction wheel is periodic, $\tau = \tau _0 \sin {\Omega t}$, a limit cycle exists in the reduced velocity space^40^ and the sleigh moves along a serpentine path with its time averaged path converging to a straight line illustrated by a sample result in Fig. 2a.

### Limit cycles in reduced velocity space of a hydrofoil

We consider a hydrofoil modeled as a NACA 0018 symmetrical airfoil that contains an internal reaction wheel with a moment of inertia $I_r$ and angular velocity $\Omega _r$. The oscillatory motion of the reaction wheel generates a periodic torque on the hydrofoil given by $\tau = -I_r\dot{\Omega }_r$. We simulate its motion due to a periodic torque $\tau$ using a vortex panel method. Panel methods are a form of computational fluid dynamics (CFD) that relies entirely on potential flow theory with point and line vortices. In these methods boundary (structure) surfaces are decomposed into discrete *panels* with source and vortex distributions on each panel such that flow does not pass through the surface. While the lack of viscous effects can reduce simulation fidelity compared to modern meshed Navier–Stokes solvers, panel methods have lower computational cost and easily incorporate body movement due to the lack of meshing. For these reasons, panel methods continue to be the preferred simulation tool for high Reynolds number fluid interaction problems^41,42^, where the flow is largely dominated by inertial effects which are easily captured by the panel method, and the neglected viscous effects are comparatively insignificant. This includes the dynamics of swimming of large and/or fast swimmers, where experimentally validated panel methods have long been used from quantifying the efficiency of flipper shapes^43^ or flapping airfoil kinematics^44^ and explaining the flow dynamics of full fish models^45^. Though the decreasing cost of computing power has lead to increasing use of meshed finite volume techniques in swimming problems, new panel methods are still in development^46^, and they are still widely used for swimming problems, including anguilliform^47^ and cetacean^48^ swimming.

In a vortex panel method, each of the *N* panels has a source distribution of strength $\sigma _i$, which varies between panels, and a vortex distribution of strength $\gamma$ which is constant over the body. The Neumann boundary condition is applied, which stipulates that no flow passes through a panel midpoint, or $\langle u_i , \eta _i \rangle =0 \; \forall \, i \in \{1,\ldots ,N\}$, where $u_i$ is the flow velocity vector at the center of panel *i* relative to the body, and $\eta _i$ is the surface normal unit vector at the same control point. The Kutta condition enforces the condition that static pressure *p* is continuous at two panel midpoints adjacent to the tail, i.e., $p_1=p_N$. To enforce this condition, vortex shedding occurs at the tail, by calculating the change in circulation about the body at every time step and applying an opposite circulation to a *wake panel* at the tail, which is then shed as a point vortex of equal circulation at its center. The structure of the system allows $\gamma$ to be solved independently, and because the flow velocity due to a specific source or vortex panel is linear in its unknown strength, the *N* unknown $\sigma _i$ values can be found by solving the linear system of *N* equations arising from the Neumann boundary condition. With $\gamma$ and the $\sigma _i$ found, the flow field is fully determined, and the pressure distribution around the body can be computed from the unsteady Bernoulli equation. This is implemented by calculating the velocity potential at each point on the body relative to the leading edge via a path integral of fluid velocity along the body, neglecting circulation. The time derivatives of these potentials are calculated by finite differences. The moving potential reference point results in a value that varies with time but is equal across the body added to all of the pressures, but because of body closure, this value has no effect on the calculated forces or moments. From the pressures, the resultant torques and forces acting on the body can be computed. An additional linear dissipation force is applied on each body degree of freedom linearly proportionally to the velocity, which prevents drag-free gliding that would otherwise be possible without skin friction. A snapshot from a sample simulation of the motion of the vortex wake due to a periodic torque on a Joukowski foil is shown in Fig. 2b. The panel code was validated in steady flow by comparing computed lift coefficients to known experimental values for a range of angles of attack^49^. It was also validated in unsteady flow^34^ where it was compared to a rotor-driven swimming hydrofoil experiment, and it was found that the swimming model results in similar trajectories to the experimental system.

## Parameter estimation for the Surrogate model

The similarity of the limit cycles of the Chaplygin sleigh and the hydrofoil and their trajectories in the plane, in response to periodic control input (torque) together with the similar nonholonomic constraints on the both the systems, motivates the use of the Chaplygin sleigh as a surrogate model for the swimming foil. The limit cycles of the Chaplygin sleigh in the reduced velocity space depend on the parameters $p=(m,I,b,c_u,c_{\omega })$. For an accurate surrogate model, these parameters need to be chosen such that resultant limit cycles due to periodic torques are nearly the same (with the same mean value and amplitudes) as those of a hydrofoil. This is accomplished by first gathering data from simulating the motion of the hydrofoil, with each simulation being 100 seconds long with data acquired at time increments $dt=0.1$ with forcing $\tau =A \sin {t}$. Two different models are fit, one where $A=1.0$ and another where $A=1.2$, and the sensitivity of the results to the forcing amplitude used to fit the surrogate model is explored. The surrogate model fit from simulations using the lower forcing amplitude resulting in a smaller $u_0 = 0.606$ (of the swimmer) will be referred to as the “low speed surrogate model”, while the latter model fit from simulations using the higher forcing amplitude with a higher $u_0 = 1.90$ will be referred to as the “high speed surrogate model”. For each time step, the current state $s = (u, \omega )$, action $\tau$, and next state $s^{+1} = (u^{+1}, \omega ^{+1})$ are stored in buffer *B*. An optimization routine is then performed to minimize the least squares error

11 $$\begin{aligned} p_0 = \arg \min _{p} \sum _B\left( \left( u+ \left( b \omega ^2-\frac{c}{m} u \right) dt - u^{+1} \right) ^2 + \left( \omega + \left( \frac{\tau -m b \omega u}{I+m b^2} \right) dt - \omega ^{+1} \right) ^2 \right) . \end{aligned}$$

**Figure 3:**
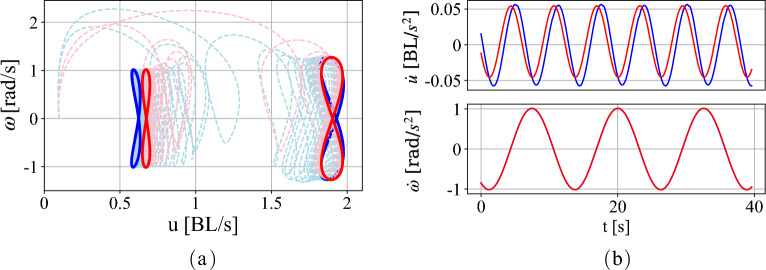
(**a**) Limit cycles of the surrogate Chaplygin sleigh (red) and the swimmer (blue) for the same periodic forcing, demonstrating convergence to similar limit cycle trajectories in the reduced velocity space. Two sets of limit cycles are shown, one due to applied periodic torque $\tau =\sin {t}$ (limit cycles on the left) and the other due to $\tau =1.2 \sin {t}$ (limit cycle on the right). (**b**) The vector field of the governing equations for the surrogate Chaplygin sleigh (red) and the swimmer (blue) at the lower velocity. The units shown are for the simulated swimmer, the Chaplygin sleigh states are dimensionless.

Figure 3a shows the trajectories of the swimmer (blue) and the surrogate model (red) obtained from (11) for the low speed model (on the left) and the high speed model (on the right). In both the cases the surrogate models’ trajectories in the reduced velocity space converge to limit cycles that are qualitatively similar to those of the swimmers. In the pair of limit cycles on the left, the apparent mismatch of the limit cycles is largely due to an error (about $10\%$) in the mean value of the speed $u_0$. This is evident in Fig. 3b which shows the evolution (in red) of $\dot{u}$ and $\dot{\omega }$ for the low speed surrogate model. The evolution of these state variables for the hydrofoil are also shown in Fig. 3b in blue. The error in the angular velocity (and its derivative) is negligible between the hydrofoil and its nonholonomic surrogate model and the error in $\dot{u}$ is small, with a value of 0.021 for the low velocity surrogate, which results in an error in mean translational velocity $u_0$ of 0.066. Error in $u_0$ for the higher velocity surrogate model is significantly lower, at $3.11 \times 10^{-5}$.

Here we note that the surrogate modeling fits the parameters of a three degree of freedom rigid body model to approximate the complex high-dimensional interaction with the vortex wake of a hydrofoil. Therefore, the mapping from the dynamics of a swimmer to that of a Chaplygin sleigh cannot be unique. The same surrogate model can be mapped non-uniquely to the dynamics of different swimmers. For example, for the low speed surrogate model, the dimensionless parameter values are found to be $m=0.93$, $I=0.98$, $b=0.068$, $c_u=0.041$, and $c_{\omega }=0.0043$. For the high speed surrogate model, the corresponding parameters are $m=0.36$, $I=0.93$, $b=0.17$, $c_u=0.026$, and $c_{\omega }=0.058$. Moreover, the error between the dynamics of the surrogate model and the true swimmer it models, increases with changing the velocity or forcing. We use both of these surrogate models in the subsequent curriculum learning to demonstrate that the qualitative similarity between the physics (mainly that efficient motion lies on an invariant manifold) is more important to the training than quantitative similarity, as the two surrogate models have quantitatively dissimilar parameters.

## Reinforcement learning

Control for fish robots is challenging due to the high complexity of the fluid–body interaction and the need for periodic motion. The most popular current approach is based on the central pattern generator (CPG), a neural assembly found in vertebrates that has periodic outputs which provide the rhythm for periodic locomotion^50^. Artificial attempts to reproduce this functionality often determine the deflection of each actuator as an oscillator, where parameters determine the amplitude, frequency, and relative phases of the actuators^51^. While this does typically result in swimming behavior, the exact oscillator parameters are typically chosen heuristically, and though formal parameter optimization can result in large improvements in swimming speed, they must be performed by algorithms such as particle-swarm optimization that select parameters to optimize the reward over entire trajectories in an open-loop fashion^52^. Reinforcement learning has emerged as a more efficient way to optimize controller parameters than pure trajectory optimization because it can utilize knowledge about intermediate states within the trajectory to improve the controller (policy), provided that the control action is only a function of the states and controller parameters. Here, we teach the controller to perform oscillations based on feedback using a curriculum, instead of encoding the oscillator directly into the controller architecture. This allows parameter optimization to be performed on comparatively few trajectories of the simulated swimmer.

We apply a Deterministic Policy Gradient (DPG) algorithm with curriculum learning to control the foil in the panel method simulated swimming environment to track a reference path at a reference velocity. This path tracking algorithm is decomposed into two parts: a pure pursuit algorithm^53,54^ that determines a target turning angle given the path geometry, and a DPG-trained actor that performs the specified turn at the desired velocity.

The pure pursuit algorithm is the simpler of the two: given a sequential list of points (*X*, *Y*) defining a path, a list of vectors ${\bar{{\textbf {r}}}}$ spanning from the center of the pursuer to each point can be constructed, and a target point $(X_i,Y_i)$ with vector ${\bar{{\textbf {r}}}_i}$ can be selected with *i* initialized at 0. At every time interval, all vectors ${\bar{{\textbf {r}}}}$ are reconstructed based on the current pursuer position, and if ${\bar{|{\textbf {r}}}}_{i}|< d$, where *d* is a constant which specifies the sight horizon, then *i* is increased iteratively until the inequality becomes false. The vector $\bar{{\textbf {r}}}_{i}$ is then taken as the pursuit vector, and the angle of ${\bar{{\textbf {r}}}_i}$ in the fixed frame is recorded as ${\theta_{target}}$, and in the local frame the error between the current and desired heading is defined as ${\theta_e}=\theta_{target}-\theta$. In the limit as $(d, \theta_{e}) \rightarrow 0$ and for a continuous path (*X*, *Y*), the tracking error goes to zero. Such a pursuit algorithm was implemented for path tracking by a Chaplygin sleigh in Fedonyuk^35^. The trajectory $(x(t), y(t), \theta (t))$, of the Chaplygin sleigh in the plane when the torque is periodic is serpentine, but the time averaged trajectory converges to a straight line. If the sight horizon *d* is too small, i.e. $(\frac{d}{u_r} \ll \frac{1}{\Omega })$ for a reference velocity $u_r$ and stroke frequency $\Omega$, the pursuit vector ${\bar{{\textbf {r}}}_i}$ and $\theta _{target}$ oscillate rapidly to direct the body back to the path, which interferes with the agent’s derived stroke frequency. In contrast, a control method chasing a more distant point tends to smooth the small-scale features of the path and minimize oscillations of the trajectory, but where the desired path is sharply curved, track a chord cutting through the required path. Here we take $d=10$ body lengths, such that $d \sim \frac{u_r}{\Omega }$ for speeds considered, which we find to be appropriately small to capture the details of the paths considered, while not small enough to interfere significantly with the frequency of oscillations.

### Curriculum learning

The curriculum learning has three training steps: a supervised step of pre-training the policy to model a given control on the surrogate Chaplygin sleigh, a DPG training step to optimally control the surrogate Chaplygin sleigh model, and a third step transferring the same trained model into the fluid–robot simulation environment for further training.

**Figure 4:**
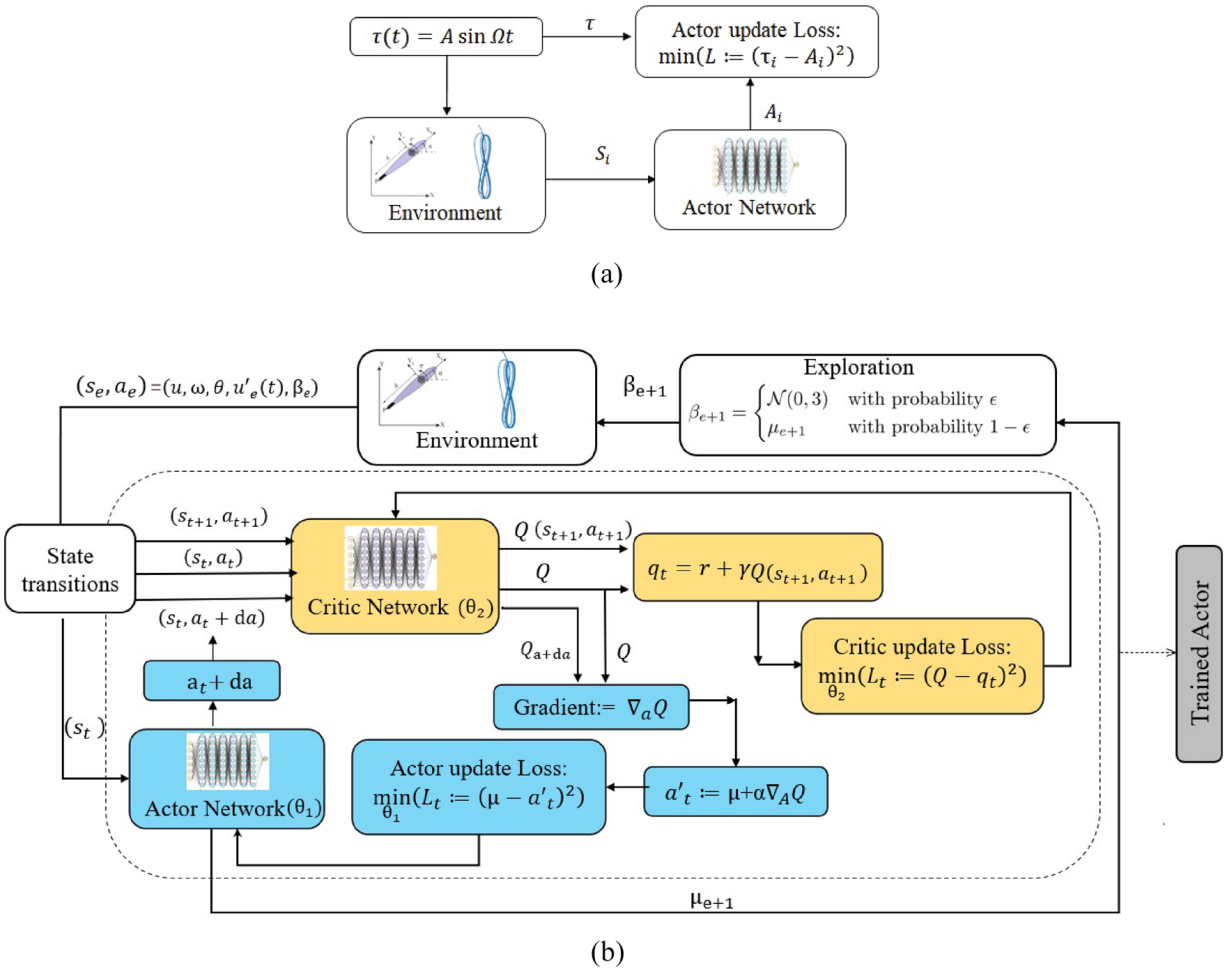
(**a**) A schematic of pre-training to encode the limit cycle features of the reduced velocity space and periodic gaits into the policy output of an actor. (**b**) A schematic illustration of the application of the modified DPG algorithm to train a policy that can make the surrogate Chaplygin sleigh track a limit cycle and heading angle.

**Figure 5:**
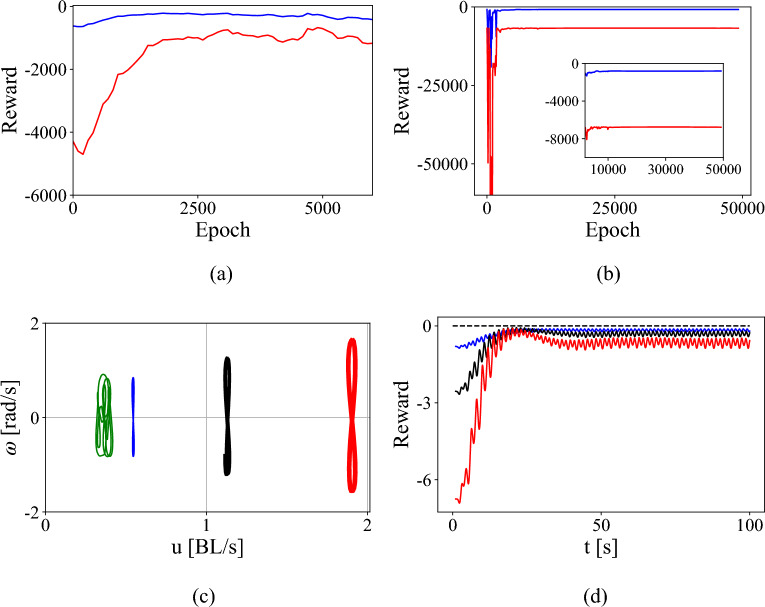
Total reward during epochs of training on the low-speed Chaplygin sleigh surrogate model for (**a**) the pre-trained actor and (**b**) an actor without pre-training. The red and blue lines show the reward while learning a policy to track a high speed ($u_t=2.5$) versus a low speed ($u_t=0.8$) respectively. (**c**) Limit cycles resulting from the policy learned on the surrogate model for target velocities of 0.8, 1.5 and 2.5 (blue, black, and red respectively) as well as the policy before training (green), and (**d**) The reward function for this policy in each case.

The supervised step of pre-training the sleigh to model a given control on the Chaplygin sleigh imprints into an actor a known control algorithm as an initial policy from which further exploration can enable the actor to learn policies for other reward functions (control goals), as will be seen by results in Fig. 5 in the following section. The first supervised step is inspired by previous work to control the surrogate Chaplygin sleigh, such as^35^ in which velocity tracking with a purely sinusoidal input and in^55^ where steering with a purely proportional control was investigated. Initializing the actor to perform a superposition of these control methods with arbitrarily chosen constants is a better starting point than a fully random actor. The initial control function is selected as

12 $$\begin{aligned} \tau =0.3 \sin {t} -0.05 \theta _{e}, \end{aligned}$$

which, with constant $\theta _{target}$ converges to a limit cycle and ${\bar{\theta }_{e}} \xrightarrow {} 0$ and $u_0 \xrightarrow {} 0.32$ for any initial conditions $(u(0), \theta _e (0))$ in a neighborhood of zero. This pre-training control function is arbitrary and any other target velocity within a pre-defined feasible range $u_0 \in [0, u_{max}]$ could have been achieved without affecting the subsequent algorithm. The pre-training generates a deterministic policy $\mu (s|\theta _1)$ with weights $\theta _{1}$ and no explicit time dependence that generates a similar periodic gait to that generated by the time-dependent prescribed forcing in (12). This is achieved by collecting state and action vectors (*s*, *a*) of a trajectory using the latter time-dependent policy, and finding $\theta _1$ (the network architecture corresponding to these weights is explained in Section “Deterministic policy gradient for tracking limit cycles and heading angle by Chaplygin sleigh”) that minimize the sum of the error $(a-\mu (s|\theta _1))^2$ over the points sampled from the trajectory using stochastic gradient descent. The chosen trajectory has length $t=100$ seconds at intervals $dt=0.1$s. This optimization is done in 5 epochs which prevents over fitting, which is particularly important as data is used from only one trajectory. A schematic of this pre-training procedure is shown in Fig. 4a.

### Deterministic policy gradient for tracking limit cycles and heading angle by Chaplygin sleigh

Knowing $\theta _{e}$ and prescribing a target velocity $u_{t}$, the optimal control problem can be reduced into a regulation problem: find a policy $\mu (u,\omega ,\theta _{target},u_{t})$ such to maximize the cumulative reward

13 $$\begin{aligned} r=-(u-u_t)^2-0.2 |u-u_t|-0.5 (\theta _e)^2 -0.1 |\theta _e| \end{aligned}$$

which can otherwise be denoted as

14 $$\begin{aligned} \arg \max _{\mu } \int _{t_0}^{t_f} r(u,\omega ,\theta _{e},u_{t}) dt \end{aligned}$$

regulating $u \rightarrow u_t$ and $\theta _e \rightarrow 0$. The policy $\mu (u,\omega ,\theta _{e},u_{t})$ is the same as the (control) torque $\tau$ with state feedback of $(u,\omega ,\theta _{e},u_{t})$. Though $u_t$ is not strictly a state of the system and does not have any dynamics except for those prescribed to it (variable speed tracking for instance), it must be relayed to the policy together with the states to allow appropriate control, so from the perspective of the DPG algorithm it is a state, and we will henceforth refer to it as such. This reward function is designed to simultaneously minimize the error in angle and velocity tracking, which are mutually exclusive goals: maintaining the target velocity requires “flapping” motion that results in periodic deviation from the desired heading angle. While the $L^2$ norm is traditionally used in cost functions for regularization problems and regression, it penalizes large deviations from the target value much more harshly than small ones, which makes it impossible to select a weight on the angle tracking reward that will adequately penalize small biases, while not penalizing flapping oscillations too much, which would result in swimming speeds well below the velocity target. However, allowing small biases to exist in the tracking angle can cause the swimmer to deviate from the prescribed path over time. This problem is alleviated by introducing an $L^1$ term on the angle tracking error to penalize those small biases. Similarly, an $L^1$ term is included in the velocity tracking reward to reduce the small negative bias in the velocity that results from the oscillation magnitude-velocity tradeoff. Specific weights were tuned on the surrogate model by repeated trials.

Approaches to this and similar problems include using an analytical harmonic balance calculation to solve the inverse problem of finding a biased sinusoidal input capable of reaching desired limit cycles for the Chaplygin sleigh such as in^40^. An extension for the case of a hydrofoil tracking a limit cycle was shown in^34^ but this approach cannot be extended for path tracking or tracking variable velocities. The approach in^55^ successfully uses proportional control on the heading angle, but there is not clear way to expand this approach to simultaneous velocity tracking. In the absence of a standard control method for even the path tracking problem for the Chaplygin sleigh, and the associated difficulty in carrying a similar approach to a swimmer we consider this problem well suited for reinforcement learning. Since the action space (allowable control) $\tau$ is continuous, DPG has the ability to utilize the entire action continuum^36,37^, which makes it suitable for this problem. Our DPG implementation is explained graphically in Fig. 4b, and explained in text below.

Denote $s=(u,\omega ,\theta _e,u_t)$ and define the policy $\mu (s)$ as a neural network with weights ${\textbf {w}}$. The reward maximization problem can be approached with any sufficiently general numerical optimization algorithm, by perturbing the weights of $\mu$, then recalculating the trajectory and its associated cumulative reward. While this Monte–Carlo approach will typically arrive at a maximum, it requires many trajectory iterations and an extensive training process, which combined take a long time to achieve convergence to any optimal policy. Policy gradient methods make this much faster by also calculating the value of a given state-action pair using a *critic* network *Q*(*s*, *a*). Quantifying the value of an action in the context of a specific state allows for specific updates to the policy to perform higher-value actions, which requires fewer trajectories than the Monte–Carlo approach because there is only one cumulative reward per simulation, but many actions, generating more data that allows for finer training. In simple terms, it is more effective to promote and curtail specific actions than it is to promote or curtail a set of weights based on the value of its average action.

In a traditional policy gradient algorithm, the policy $\pi$ is a probability distribution that estimates the appropriate action out of a discrete pre-determined set. While this is effective in many benchmark problems that feature discrete (and often small set of) actions^56,57^ or more commonly in games with discrete on/off controls^58^, it is a limitation where a continuous spectrum of allowable actions is available. DPG has the ability to utilize the entire action continuum^36,37^, which makes it an intuitive choice for this problem.

More formally, the traditional DPG algorithm features two function approximators: an *actor* $\mu _{\theta 1}(s)$ and a *critic* $Q_{\theta 2} (s,a)$ where $\theta _1$ and $\theta _2$ are weights that define the approximation. The approximators used with this algorithm, such as in^37^, are typically neural networks due to their utility as universal function approximators^59^ and the ill-suitedness of competing discrete approximators, such as tables, to a continuous state and action space. These weights are typically initialized in a Gaussian distribution, and an empty experience buffer *R* is also generated. We use an $\epsilon$ *greedy* exploratory policy $\beta (s)$, which when sampled has a $1-\epsilon$ chance of returning $\mu (s)$ and an $\epsilon$ probability of returning a random value pulled from a normal distribution with deviation $\sigma$. The environment is simulated and the results stored in the experience buffer in the form $(s,r,a,s^{+1})$, where $s^{+1}$ is the next state which is transitioned into. The discounted expected future reward *Q*(*s*, *a*) can be updated to equal the sum of the current reward and the discounted approximated future reward for the known next state, a process known as *bootstrapping* which can be written as

15 $$\begin{aligned} Q_{target}(s,a)=r+\gamma Q_{\theta _2}(s^{+1},\mu _{\theta _1}(s^{+1})), \end{aligned}$$

where $\gamma$ is the discount factor. A new set of actions can then be selected that maximize expected reward by performing one step of gradient ascent:

16 $$\begin{aligned} \mu _{target}(s)=\mu _{\theta _1}(s)+\alpha \frac{\partial Q_{\theta _2}(s,a)}{\partial a} \end{aligned}$$

where the gradient can be computed efficiently along one dimension by a finite difference approximation

17 $$\begin{aligned} \frac{\partial Q_{\theta _2}(s,a)}{\partial a} \approx \frac{Q_{\theta _2}(s,a+d a)- Q_{\theta _2}(s,a-d a)}{2 d a}. \end{aligned}$$

This gradient can also be computed by automatic differentiation, which is more efficient than finite difference methods for higher-dimension action spaces. Updating both networks $Q_{\theta _2}(s,a) \rightarrow Q_{target}(s,a)$ and $\mu _{\theta _1}(s,a) \rightarrow \mu _{target}(s,a)$ is then a supervised learning problem, where we minimize the least-square-error using 5 epochs of the *adam* algorithm^60^, which is a stochastic gradient descent algorithm with momentum. This supervised approach is a departure from the original algorithm, which instead updates both sets of weights with a single step of gradient descent; we find that higher optimization speed of the *adam* algorithm compared to deterministic gradient descent makes this supervised approach faster on this problem. We use the *adam* parameters recommended in page 2 of^60^, except with $\varepsilon =10^{-7}$. These updates are repeated *n* times before a new batch of *m* environment simulations are generated and appended to the experience buffer, ensuring that there is enough data about perturbations local to the current trajectories to allow local optimization.

We selected initial parameter values of $n=10$, $m=10$ for the Chaplygin sleigh training, and $n=10$ and $m=1$ for the fluid simulation training. Additionally, we used values of $\alpha =0.01$, $\gamma =0.99$ $\sigma =3$, $a=10^{-5}$, and an experience buffer of size up to $3 \times 10^5$. As the training progresses, the values of $u_t$ for each simulations are drawn from a widening probability distribution. At first, training is performed with $u_t=1$. After 500 iterations, the target velocity for each trajectory is drawn from a uniform distribution such that $0.8 \le u_t \le 1.2$, and the target velocity is held constant within a trajectory. This is broadened to $0.6 \le u_t \le 1.6$, $0.4 \le u_t \le 2.0$, and $0.2 \le u_t \le 3.0$ after 1000, 1500, and 2500 iterations, respectively. This gradual introduction of different target velocities serves as another layer of curriculum, and reduces the risk of non-convergence. One modification is made to the DPG algorithm described thus far: because the critic is initialized with random weights but the actor is pre-trained, actor updates are disabled by defining $\alpha =0$ for the first 10 iterations, at which point the critic has grown consistent with the actor and the learn rate can be reset to $\alpha =0.01$.

When the episode reward on the sleigh environment qualitatively is seen to reach a peak or asymptotic convergence to a solution, the actor and critic are transplanted to the fluid simulation. The states used as policy inputs remain the same, however in the Chaplygin sleigh they correspond to full state feedback; in the simulated swimmer, the limited state feedback is insufficient to even fully describe the motion of the rigid body, let alone the high-dimensional fluid. This lack of observability makes training on the swimming simulations more data expensive, as the underlying Markov assumption that every state-action pair has a fixed probability distribution of states that it will transition to is not accurate. Though the overall system is Markov in that a complete state-action pair does transition deterministically to a specific next state, because the “state” given to the policy is a small subspace of the true state, it appears as if the system is non-Markov from the perspective of the agent. This reduced data efficiency, combined with the increased computational cost of generating data, makes training very slow in terms of wall-time.

Dense neural networks are the most popular choice of function approximator for DPG. A dense network contains one or more layers of neurons, and the value of each neurons is computed as a weighted sum of the neurons of the previous layer, with different weights for each neuron. The values are then passed through an activation function before being passed to the next layer, where the process is repeated until it reaches a neuron that is read as the output. We selected a 6-layer dense neural network, featuring an input layer of size 3 for the actor and size 4 for the critic, 5 layers of 40 neurons each with rectified linear unit (ReLU) activation, and a single output node with linear activation. In the policy a function $\tau =\tau _m \tanh {\frac{a}{\tau _{m}}}$ was used to smoothly attenuate the prescribed output *a* to a bounded torque value $\tau$, which improves convergence. We select $\tau _m=4$ as the range of allowed forcing, which we find to be large enough to perform effective control but small enough to not cause numerical problems in the fluid simulation.

The reinforcement learning uses an in-house implementation of DPG in Python with Tensorflow, accessed through the Keras API. Both of the neural networks are implemented in Keras, and the supervised updates of both the actor and critic networks are performed with *Model*.*fit*(), the Keras supervised learning utility, on batches of 1000 state transitions from the experience buffer. Both environments are implemented in Python with state transitions saved at interval $\Delta t = 0.1$ s. The sleigh simulations are trained with an $\epsilon$*-greedy* exploration strategy with $\epsilon =0.2$. The fluid simulations instead follows a Gaussian exploration approach, where normally distributed noise of deviation 0.2 is added to every action during exploration, which reduces the large discontinuities in torque that are seen in $\epsilon$*-greedy* exploration, because they in turn cause large magnitudes of shed vorticity which results in an unrealistic wake.

## Results: velocity and path tracking

The supervised step of pre-training the sleigh to model a given control on the Chaplygin sleigh imprints into an actor a known control algorithm as a starting policy from which further exploration can enable the faster learning of policies for other reward functions (control goals). Figure 5a shows the reward during training by the low speed surrogate model that has been pre-trained to output periodic control actions [given in Eq. (12)]. The red curve shows the reward while the agent is learning a policy to track a high speed $(u_t = 2.5)$ while the blue graph is for tracking a low speed $(u_t=0.8)$. Figure 5b shows the reward function during training of an actor without pre-training to a sub-optimal policy and instead using an actor network with weights initialized to Gaussian noise.

The pre-training torque has an amplitude of only 0.3 while the torques required to track the low and high speed velocities are 1 and 1.2 respectively. Despite pre-training with such a suboptimal policy, the pre-trained actor learns faster and better, with the reward converging to higher values $-350$ (blue) for lower speeds while the reward for the agent without pre-training converges to $-800$ while also taking more epochs.

The limit cycles generated in the reduced velocity space as a result of this trained policy are shown for three different target velocities $u_{t} = (0.8, 1.5, 2.5)$ in Fig. 5c along with the limit cycle produced in pre-training where $u_0 = 0.32$. It can be seen that the actor adapts from generating a quazi-periodic trajectory to generating consistent limit cycles near the desired velocities. It also becomes more efficient at converting rotation into translation; the blue (trained) and green (untrained) trajectories have similar maximum amplitudes of $\omega$, but the trained trajectory achieves a higher velocity *u*.

The mean value of the velocity *u* for the limit cycles shown in Fig. 5c have an error that does not decay, due to the fact that the reward function is a sum of the rewards accrued by minimizing the error $u-u_{t}$ as well as the error $\theta _{e}$. The serpentine motion of the sleigh results in a necessary non-zero error in the heading angle, which can be reduced by smaller amplitude torques which slows down the sleigh. The learned policy, with the reward shown in Fig.5d, is a compromise between achieving $u_{t}$ and minimizing the error in the heading angle $\theta$.

**Figure 6:**
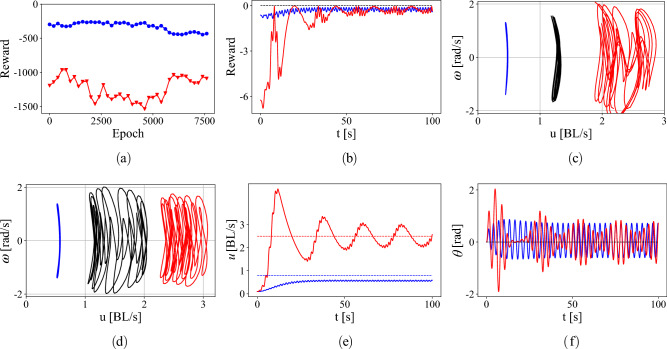
Trajectories on the hydrofoil after training on a low speed surrogate model actor. (**a**) Reward function during the epochs of training on fluid-hydrofoil simulations and (**b**) reward while executing the optimal policy tracking velocities and heading angle starting from rest. (**c**) Trajectories in the reduced velocity space due to the optimal policy of an actor trained on just the surrogate model and (**d**) produced by the optimal policy by an actor trained on additional fluid-hydrofoil simulation. (**e**) Velocity tracking by the hydrofoil for two tracking velocities and (**f**) simultaneously tracking 0° heading angle. Color legend—tracking speed $u_t = 0.8$ blue , $u_t = 1.5$ black and $u_t = 2.5$ red.

In the next stage of the curriculum learning, the agent trained on the surrogate Chaplygin sleigh is transferred to train in the fluid-hydrofoil simulation environment using the panel method, for the velocity and heading angle tracking problem with the aim of improving the sub optimal policy of the surrogate sleigh actor. A total of 150 trajectories of the swimmer and 7500 DPG iterations were used to adapt the policy of the Chaplygin sleigh to the swimmer, at a rate of 16.1 mins per 1000 DPG updates and 5.49 mins per trajectory generated on an Intel Xeon Gold 6148F processor. For comparison, a batch of 10 Chaplygin sleigh trajectories can be generated in 6.1 s. As a result, the training time with the curriculum is much smaller than what would usually be required if the swimmer were to be directly trained without the intermediate training on the surrogate Chaplygin sleigh model. Figure 6a shows the reward function during each epoch using the low speed surrogate sleigh model to track a high (red) speed of $u_t = 2.5$ and low (blue) speed of $u_t = 0.8$.

The reward at the beginning of the first epoch is due to the optimal policy learned on the surrogate sleigh. The reward during the training on the fluid–hydrofoil interaction simulation does not change significantly. However this seeming lack of learning is deceptive, as borne out by examining the velocity of the hydrofoil. Figure 6c shows the trajectories in the reduced velocity space (ignoring a 50 second transient solution) for a swimmer produced by the optimal policy of the actor trained only on the surrogate sleigh model while Fig. 6d shows the same trajectories for a swimmer produced by the optimal policy of the actor trained further on the fluid–hydrofoil simulations. The trajectories in the reduced velocity space visit a larger range of *u* values after training on the fluid simulation. While the velocity of the swimmer does not converge to the target velocities, it does oscillate with a mean value close to the target velocity, when the target velocity is high (red) as shown in Fig. 6d. This strategy forgoes the compromise between oscillation angle and velocity seen in the Chaplygin sleigh training, and instead features intervals of high-torque, low-reward periods of acceleration, followed by high-reward periods of low-torque coasting. The low-frequency periodic component of the velocity and pitch angle for the high-velocity tracking in this swimming strategy are shown in Fig. 6e and f, respectively. This style of ‘burst-and-coast’ swimming is frequently observed in fish^61^, and is consistent with other results in the RL swimming literature^25^. This strategy does not emerge during training on the surrogate model, but does emerge for high swimming velocities after transfer to the swimming simulation, even before continuing training. The continued training on the fluid simulations expands the range of target velocities for which the ‘burst-and-coast’ strategy is used.

**Figure 7:**
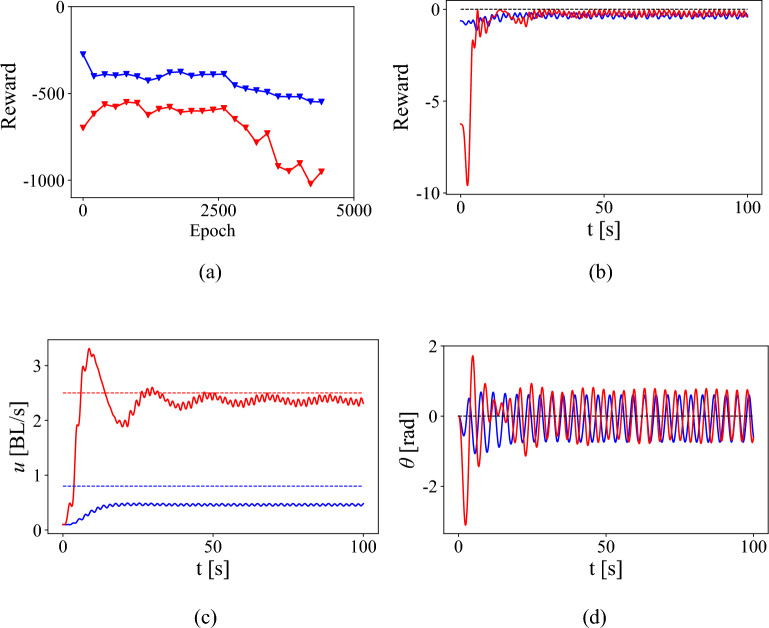
Training a high-speed surrogate model actor. (**a**) Reward function during the epochs of training on fluid-hydrofoil simulations and (**b**) the reward while executing the optimal policy tracking velocities and heading angle while starting from rest. (**c**) Velocity tracking by the hydrofoil for two tracking velocities and (**d**) simultaneously tracking 0° heading angle. Color legend—tracking speed $u_t = 0.8$ blue and $u_t = 2.5$ red.

A similar result is seen when the policy trained on the high speed surrogate model is further trained using the fluid-hydrofoil simulations to learn a policy to track different velocities and a heading angle of 0°. The reward function during the training does not increase significantly or can in fact decrease as seen in Fig. 7a. Picking the optimal policy using the actor that produces the highest epoch reward as described in Section “Deterministic policy gradient for tracking limit cycles and heading angle by Chaplygin sleigh”, produces a reward shown in Fig. 7b. The burst and coast technique is once again seen in Fig. 7c,d when tracking a high velocity (red) while a steady error is seen when tracking a lower velocity (blue).

It is to be emphasized that the lack of a significant increase in the reward function during the training on the fluid-hydrofoil simulations, compared to the reward obtained from the just the optimal policy of the surrogate sleigh actor is not a failure of the training; it can be attributed to two reasons. The first reason is that the reward function consists of a sum of terms that individually seek to minimize the error in the tracking velocity and heading angle. The oscillatory nature of the motion resulting in an oscillating heading angle, guaranteeing that the reward is always negative. The second reason is more subtle: the states *s* used in training the actor for swimming are merely a subset of the kinematic variables of the swimmer and do not include the distribution of vorticity in the fluid. The kinematics of a swimmer can be the same (or nearly so) for two different distributions of the vortex field. Moreover, an action (torque) for a given state *s* can produce a transition to completely different states that depend on the distribution of the vortex field. Since the vortex field is not an observed variable or state in the training, the state-action pair do not have a constant probability distribution, making the system seemingly non Markov from the perspective of the agent.

**Figure 8:**
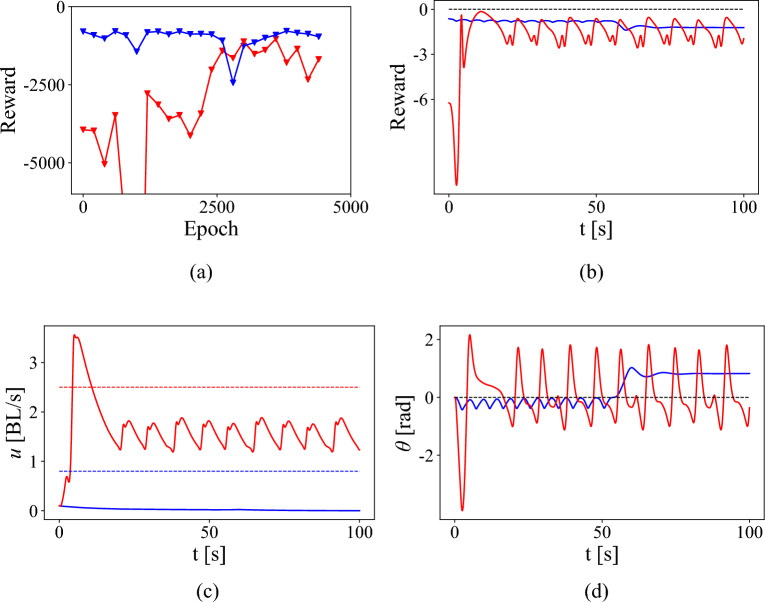
RL without surrogate model or curriculum learning. (**a**) Reward function for epoch training and (**b**) Reward function executing the optimal policy by a hydrofoil starting from rest. (**c**) Tracking low (blue) speed and high speed (red) and (**d**) simultaneously tracking 0° heading angle. For both cases, reward is lower than when trained with a curriculum. This is largely due to higher velocity error, with the swimmer tasked with reaching the low target speed instead coasting to a near-stop.

The combination of the surrogate model and curriculum learning outperforms direct reinforcement learning of a policy using fluid-hydrofoil simulations for tracking velocity and heading angle. Figure 8a shows the learning curve during such direct training, and Fig. 8b–d shows the trajectory generated by the trained policy after 4750 training iterations. The reward converges to a lower value than seen in Fig. 6a or 7a. The reward per step due to the optimal policy $\approx -3$ (shown in 8b) is also lower than the reward due to the optimal policies seen in Fig. 6b or 7b, which have a value of $>-1$ per step. This is a result of poor velocity and angle tracking, and consequently graphs of *u*(*t*) and $\theta (t)$ show large errors from the target velocities and heading angles.

**Figure 9:**
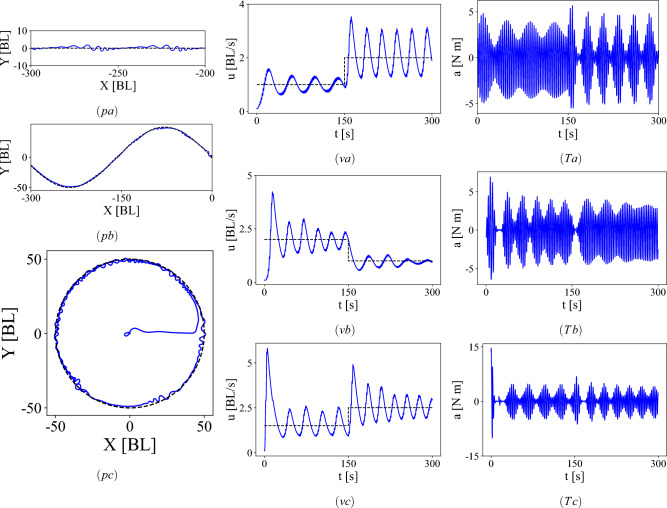
Pure-pursuit based path tracking for the simulated swimmer on (pa) a straight line and (pb) sinusoidal path and (pc) a circle. The target velocities for the case of the straight line, sinusoidal path and circle are shown by the dashed lines in (**va**), (**vb**) and (**vc**) respectively. The torques generated to track the straight line, sinusoidal path and circle are shown in (**Ta**), (**Tb**) and (**Tc**), respectively.

The last step in path tracking is to give the time dependent $\theta _{e}(t)$ determined by a pure pursuit algorithm as an input (amongst the other states *s*) to the DPG agent trained in the fluid–robot simulation environment. We show the results of three such simulations of the agent tracking a path while also simultaneously tracking a specified time-varying velocity. Figure 9 shows the swimming hydrofoil tracking (pa) a straight line, (pb) a sinusoidal path and (pc) a circle. In each case the reference velocities for the hydrofoil are shown in Fig. 9va–vc. The reference velocities are piecewise constant and either increase or decrease midway during the simulation time. The action (torque) due to the policy before attenuation by the inverse tangent in each case is shown in Fig. 9Ta–Tc. The control torques are not just sinusoidal as in the pre-training, but have significant amplitude modulation and multiple harmonics, as a result of the series of training with different models and curriculum which fine tuned the policy into a ’burst-and-coast’ strategy.

## Conclusion

Reinforcement learning methods in mobile robotics, particularly swimming robots with complex unmodelled physics, require large amounts of data covering a large subset of the relevant state space, which can be expensive and time consuming to obtain in both simulations and experiments. The results in this paper show the utility of surrogate models and curriculum-based reinforcement learning, wherein a DPG agent is trained in steps, for the control of a planar swimming robot. In each step the agent learns to generate and control certain features of the dynamics of the robot-environment action. An agent was trained to perform a series of increasingly complex tasks: tracking limit cycles in a reduced velocity space for a surrogate model, tracking heading angle and speed in the surrogate model and then finally transferred to learn doing these same tasks in a fluid–robot interaction environment. This approach reduces computational time, but more importantly creates a *physics-informed Reinforcement Learning* framework.

As a proof of concept, we have demonstrated only a limited range of curriculum on which a DPG agent can be trained. For instance, in the path tracking results in Fig. 9 the policy is trained for constant values of $\theta _{target}$, and compensates for the variable $\theta _{target}$ values prescribed by the pure pursuit algorithm with higher torque values, resulting in higher velocities and deviations from the reference velocity. Further improvement is possible by including variable $\theta _{target}$ cases in a curriculum. While the robot considered in this paper is a planar swimming oscillating hydrofoil, more ‘fish-like’ robots can be trained using a similar framework to the one proposed in this paper. Fish-like robots usually have more than one body segment in their skeleton with joints that could be elastic. Recent work, for example^62^, shows that in robots with elastic joints, tunable stiffness enables faster and more efficient swimming. Surprisingly, analogous results exist for ground-based multi-segment nonholonomic systems, see for example^63,64^, where effective stiffness tunable by periodic forcing leads to different limit cycles of varying efficiency, and multistable configurations for fast turning. Such nonholonomic systems can be used as surrogate models for multi-body fish-like robots using the same curriculum learning framework. With low-latency velocity feedback (either inertially on the body or from external measurement), the actor trained on the fluid surrogate model can be directly transplanted to the physical system, a new experience buffer can be generated, and the training can be continued on the physical system with little required setup.

This approach can be extended to three-dimensional swimming with suitable surrogate models. In three dimensions, additional control goals such as stabilization of roll and pitch may exist, while simultaneously tracking a path and/or a velocity. Both simulations of fluid–robot interactions and acquiring a large experimental data set in such cases can be even more challenging, further necessitating physics-based reinforcement learning with surrogate models. The current paper describes a preliminary proof of concept for such a framework.

## Data Availability

The datasets used and/or analysed during the current study available from the corresponding author on reasonable request.
